# Extracting Clinical Information From Japanese Radiology Reports Using a 2-Stage Deep Learning Approach: Algorithm Development and Validation

**DOI:** 10.2196/49041

**Published:** 2023-11-14

**Authors:** Kento Sugimoto, Shoya Wada, Shozo Konishi, Katsuki Okada, Shirou Manabe, Yasushi Matsumura, Toshihiro Takeda

**Affiliations:** 1 Department of Medical Informatics, Graduate School of Medicine, Osaka University, Suita, Osaka, Japan; 2 Department of Transformative System for Medical Information, Graduate School of Medicine, Osaka University, Suita, Osaka, Japan; 3 National Hospital Organization Osaka National Hospital, Osaka, Japan

**Keywords:** natural language processing, radiology report, information extraction, deep learning, machine learning, radiology, report, reports, NLP, free text, unstructured, named entity recognition, relation extraction

## Abstract

**Background:**

Radiology reports are usually written in a free-text format, which makes it challenging to reuse the reports.

**Objective:**

For secondary use, we developed a 2-stage deep learning system for extracting clinical information and converting it into a structured format.

**Methods:**

Our system mainly consists of 2 deep learning modules: entity extraction and relation extraction. For each module, state-of-the-art deep learning models were applied. We trained and evaluated the models using 1040 in-house Japanese computed tomography (CT) reports annotated by medical experts. We also evaluated the performance of the entire pipeline of our system. In addition, the ratio of annotated entities in the reports was measured to validate the coverage of the clinical information with our information model.

**Results:**

The microaveraged *F*_1_-scores of our best-performing model for entity extraction and relation extraction were 96.1% and 97.4%, respectively. The microaveraged *F*_1_-score of the 2-stage system, which is a measure of the performance of the entire pipeline of our system, was 91.9%. Our system showed encouraging results for the conversion of free-text radiology reports into a structured format. The coverage of clinical information in the reports was 96.2% (6595/6853).

**Conclusions:**

Our 2-stage deep system can extract clinical information from chest and abdomen CT reports accurately and comprehensively.

## Introduction

Radiology reports are important for radiologists to communicate with referring physicians. The reports include clinical information about observed structures, diagnostic possibilities, and recommendations for treatment plans. Such information is also valuable for various applications such as case retrieval, cohort building, diagnostic surveillance, and clinical decision support. However, since most radiology reports are written in a free-text format, important clinical information is locked in the reports. This format presents major obstacles in secondary use [1,2]. To address this problem, a system for extracting structured information from the reports would be required.

Natural language processing (NLP) has demonstrated potential for improving the clinical workflow and reusing clinical text for various clinical applications [3-5]. Among the various NLP tasks, information extraction (IE) plays a central role in extracting structured information from unstructured texts. IE mainly consists of two steps: (1) the extraction of specified entities such as person, location, and organization from the text and (2) the extraction of semantic relation between 2 entities (eg, *location_of* and *employee_of*) [6,7].

Earlier IE systems mainly used heuristic methods such as dictionary-based approaches and regular expressions [8-10]. To extract clinical information from radiology reports, the Medical Language Extraction and Encoding system [11] and Radiology Analysis tool [12] have been developed. To detect clinical terms, these systems mainly use predefined dictionaries such as the Unified Medical Language System [13] and their customized dictionaries and apply some grammatical rules to present them in a structured format.

The major issues of these systems include the lack of coverage and scalability [14]. A dictionary-based system often fails to detect clinical terms such as misspelled words, abbreviations, and nonstandard terminologies. Building exhaustive dictionaries to enhance the coverage and maintaining them are highly labor-intensive. It is also challenging to apply complicated grammar rules according to the context of the reports. In addition, IE systems based on dictionaries and grammar rules are highly language dependent and do not scale to other languages. The Medical Language Extraction and Encoding system and Radiology Analysis tool only cover English clinical texts and cannot handle non-English clinical texts. Languages other than English, including Japanese, do not have sufficient clinical resources such as the Unified Medical Language System. This has been a major obstacle in developing clinical NLP systems in countries where English is not the official language [15].

Recently, machine learning approaches have been widely accepted in clinical NLP systems [16,17]. Hassanpour and Langlotz [18] used a conditional random field (CRF) [19] for extracting clinical information from computed tomography (CT) reports. They showed that their machine learning model had a superior ability compared to the dictionary-based systems.

Deep learning approaches have drawn a great deal of attention in more recent studies. Cornegruta et al [20] built a bidirectional long short-term memory (BiLSTM) model [21] to extract clinical terms from chest x-ray reports. Miao et al [22] built a BiLSTM model to handle Chinese radiology reports. Both studies reported that deep learning approaches yielded better results than dictionary-based approaches.

Various state-of-the-art deep learning models have been applied to extract named entities [18,20,22]. Clinical systems such as concept extraction can be achieved though extracting named entities alone, whereas the relation extraction step is needed to obtain structured information about concepts and their attributes [23,24]. Extracting comprehensive information in a structured format is desirable when developing a complex system.

Xie et al [25] developed a 2-stage IE system for processing chest CT reports. They exploited a hybrid approach involving deep learning to extract named entities and a rule-based method to organize the detected entities in a structured format. They reported that their deep learning model achieved better performance, whereas the rule-based structuring approach degraded the overall performance, since the rule-based approach could not capture the contextual relations in the reports. Jain et al [26] developed RadGraph, an end-to-end deep learning system for structuring chest x-ray reports. They reported that their schema had a higher report coverage in their corpus.

In this study, we developed a 2-stage deep learning system for extracting clinical information from CT reports. For secondary use of the radiology reports, we believe that our system has some advantages compared with recent related works [18,20,22,25,26]. First, our 2-stage NLP system can represent clinical information in a structured format, which can be challenging when only using an entity extraction approach. Second, although the rule-based approach struggled to extract relations between entities in the reports [25], leveraging state-of-the-art deep learning models leads to superior performance. Third, previous studies [18,20,26] have combined clinical information about factual observations and radiologist interpretations into single entity, even though they have different semantic roles in the context. According to the context, distinct entity types are defined in our information model, which allows it to capture detailed clinical information in the reports. To structure the report more appropriately, we defined distinct entities for 2 different clinical pieces of information.

The rest of this paper is organized as follows. First, an information model was built, mainly comprising observation entities, clinical finding entities, and their modifier entities. Second, a data set was created using in-house CT reports annotated by medical experts. Third, state-of-the-art deep learning models were trained and evaluated to extract the clinical entities and relations. The entire performance of our 2-stage system was also evaluated. Finally, we evaluated the coverage of the clinical information in the CT reports using our information model.

The development of the information model was already reported in our previous study [27]. However, the previous study only focused on extracting entities and did not cover extracting relations between the entities. This study developed a 2-stage system containing entity extraction and relation extraction modules. Furthermore, although the previous study only used chest CT reports, a data set using abdomen CT reports was created in this study to validate the generalizability of our information model and 2-stage system.

## Methods

### Our Information Model

An information model was built for extracting comprehensive clinical information from free-text radiology reports. Our information model contained observation entities, clinical finding entities, and modifier entities. Observation entities are specific terms representing observed abnormal features such as “nodule” or “pleural effusion.” Clinical finding entities encompass terms such as “cancer,” including diagnoses given by the radiologists based on the observation entities. Modifier entities are subdivided into the following entities: anatomical location, certainty, change, characteristics, and size. Thus, 7 entity types were defined in our information model. A detailed description of our information model is provided in our previous study [25].

**Figure 1.**
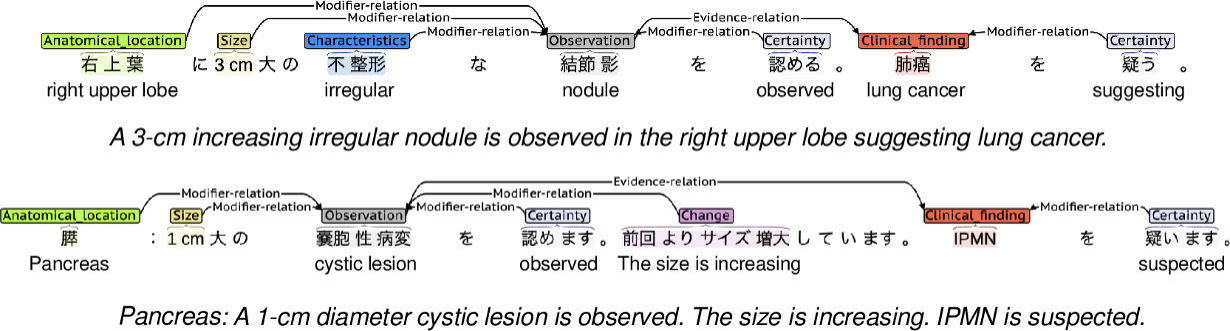
Report examples of entities and relations. IPMN: intraductal papillary mucinous neoplasm.

Furthermore, modifier and evidence relations between entities were defined. A modifier relation is derived from an observation or a clinical finding entity and a modifier entity. This relation type gives clinical information, such as the anatomical location of the observations and the characteristics of the clinical findings. An evidence relation is derived from an observation entity and a clinical finding entity. This relation is also clinically meaningful in capturing the diagnostic process of the radiologist. Report examples of entities and relations are shown in Figure 1.

### Data Set

Radiology reports from 2010 to 2021 that were stored in the radiology information system at Osaka University Hospital, Japan, were used. They consisted of 912,505 reports written in Japanese. To create a gold standard data set, 540 chest CT reports and 500 abdomen CT reports were randomly extracted. The remaining unannotated reports (911,465 reports) were used to pretrain the model.

### Ethical Considerations

This study was performed in accordance with the World Medical Association Declaration of Helsinki, and the study protocol was approved by the institutional review board of the Osaka University Hospital (permission 19276). Only anonymized data were used in this study, and we did not have access to information that could identify individual participants during the study.

### Annotation Scheme

Overall, 3 medical experts (2 clinicians and 1 radiological technologist) performed the annotation process. The gold standard data sets of chest and abdomen CT reports were developed by different annotation methods.

For the chest CT reports, the data set that was developed in our previous study was leveraged [25]. After making minor adjustments for entities, the relation types between entities were newly annotated by 2 clinicians. Following a guideline describing the rules and annotation examples, they independently annotated each report. Disagreements between the annotators were resolved by discussion. The interannotator agreement (IAA) score for the entities was 91%, as reported in our previous study [27]. To calculate the IAA score for the relations, we used Cohen κ [28], resulting in an IAA score of 81%. Both IAA scores indicated very high agreement [29].

For the abdomen CT reports, to reduce the burden of the annotation work, a deep learning model trained on the chest CT reports was implemented to preannotate the entities and relations in the reports. Annotators were provided with the preannotated reports, and they modified the result according to the guidelines. We did not compute IAA scores for the abdomen data set because it was preannotated by the deep learning model.

All entities and relations were annotated using BRAT (Stenetorp et al [30]). The number of annotated entities and relations are shown in Multimedia Appendix 1.

### Our 2-Stage System

#### Overview

**Figure 2.**
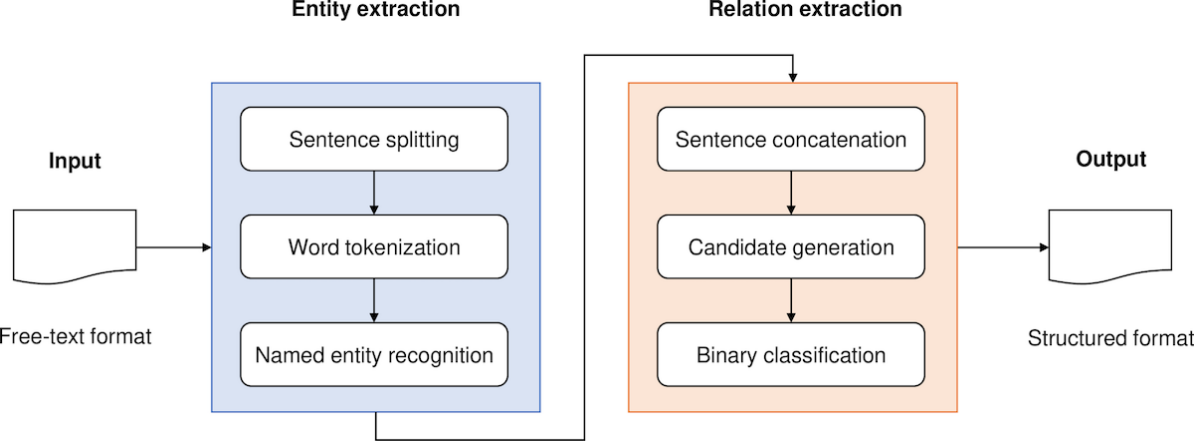
Overview of our 2-stage deep learning system.

An overview of our 2-stage system is shown in Figure 2. The system pipeline mainly consists of 2 deep learning modules. In the first step, our module extracts the clinical entities in the radiology reports according to the predefined information model. The extracted entities are fed into subsequent modules. In the second step, the relation between clinical entities is extracted. The details of each module are described in the subsequent sections.

#### Entity Extraction

**Figure 3.**
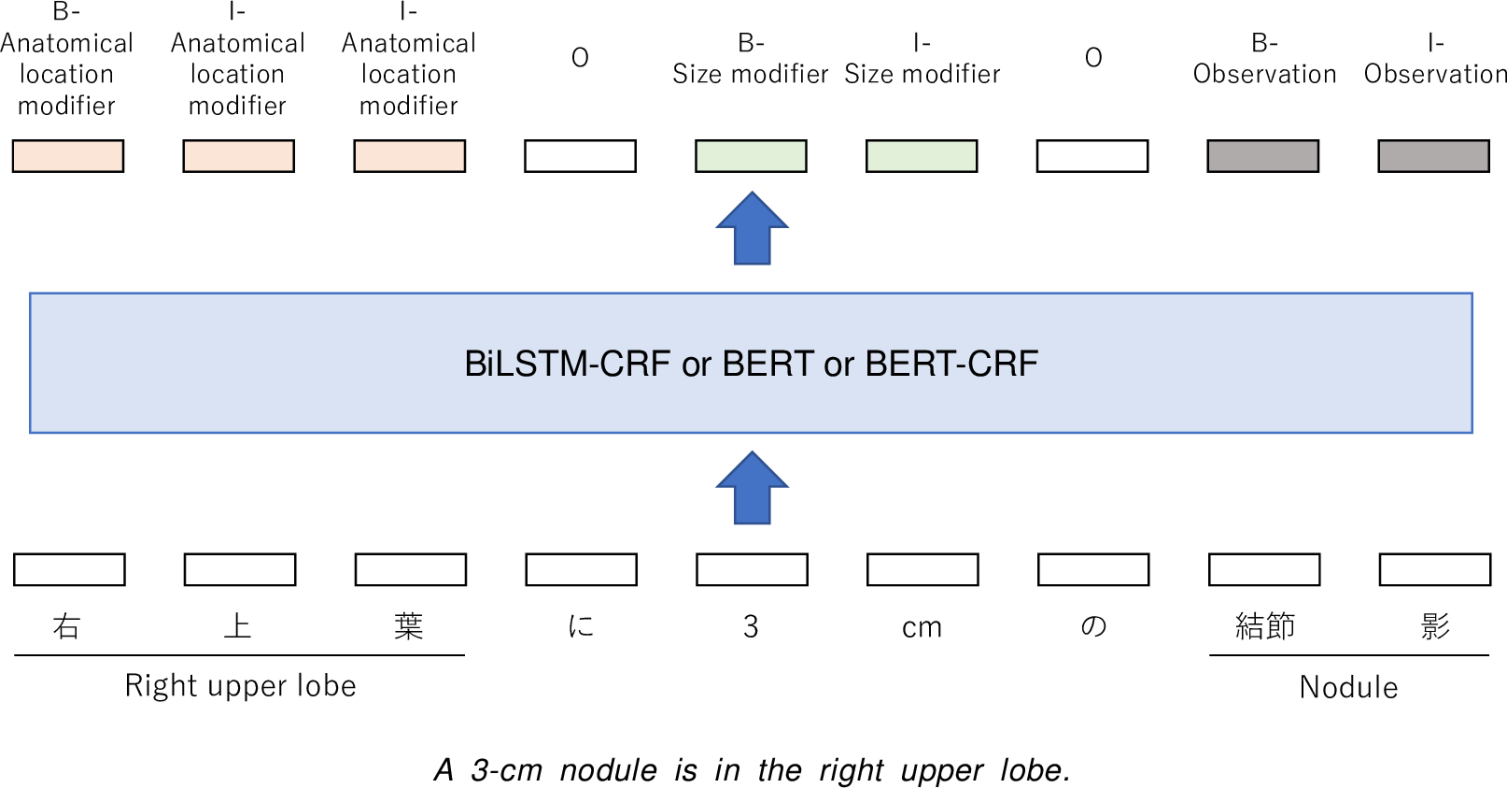
An illustration of the entity extraction module. BERT: Bidirectional Encoder Representations from Transformers; BiLSTM: bidirectional long short-term memory; CRF: conditional random field.

According to the predefined information model, this module extracts clinical entities from a report. Named entity recognition (NER) [31] is well suited for this task. As a preprocessing pipeline, the report was segmented into sentences using regular expressions, and each sentence was tokenized with MeCab (Kyoto University Graduate School of Informatics and Nippon Telegraph and Telephone Corportation’s Communication Science Research Institute) [32]. Then, a sequence of tokens was fed into the model. To represent the spans of specified entities, the IOB2 format [33], which is a widely used tagging format in NER tasks, was used. In this format, the B and I tags represent the beginning and inside of an entity, respectively, and the O tag represents the outside of an entity. A tagging example is illustrated in Figure 3.

State-of-the-art deep learning models for NER—BiLSTM-CRF [34], BERT [35], and BERT-CRF—were compared.

#### Relation Extraction

**Figure 4.**
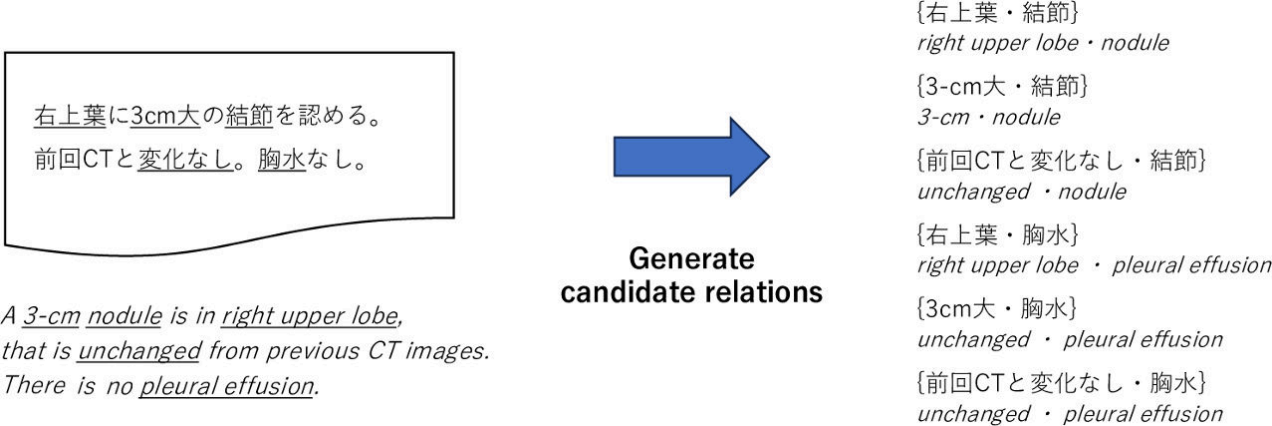
Example of instances generated for relation extraction. In this case, 6 candidate relations were generated from 2 observations and 3 modifiers. CT: computed tomography.

Following the implementation of the entity extraction module, reports with clinical entities were obtained. As a preprocessing pipeline of relation extraction, the original sentences of the report were reconstructed by concatenating sentences from the beginning to the end. This was implemented for extracting relations across multiple sentences in a report. Next, the pipeline generated possible candidate relations by each relation type in a report (see Figure 4). Then, this module solved a binary classification problem to determine the existence of relations given the candidate relations.

**Figure 5.**
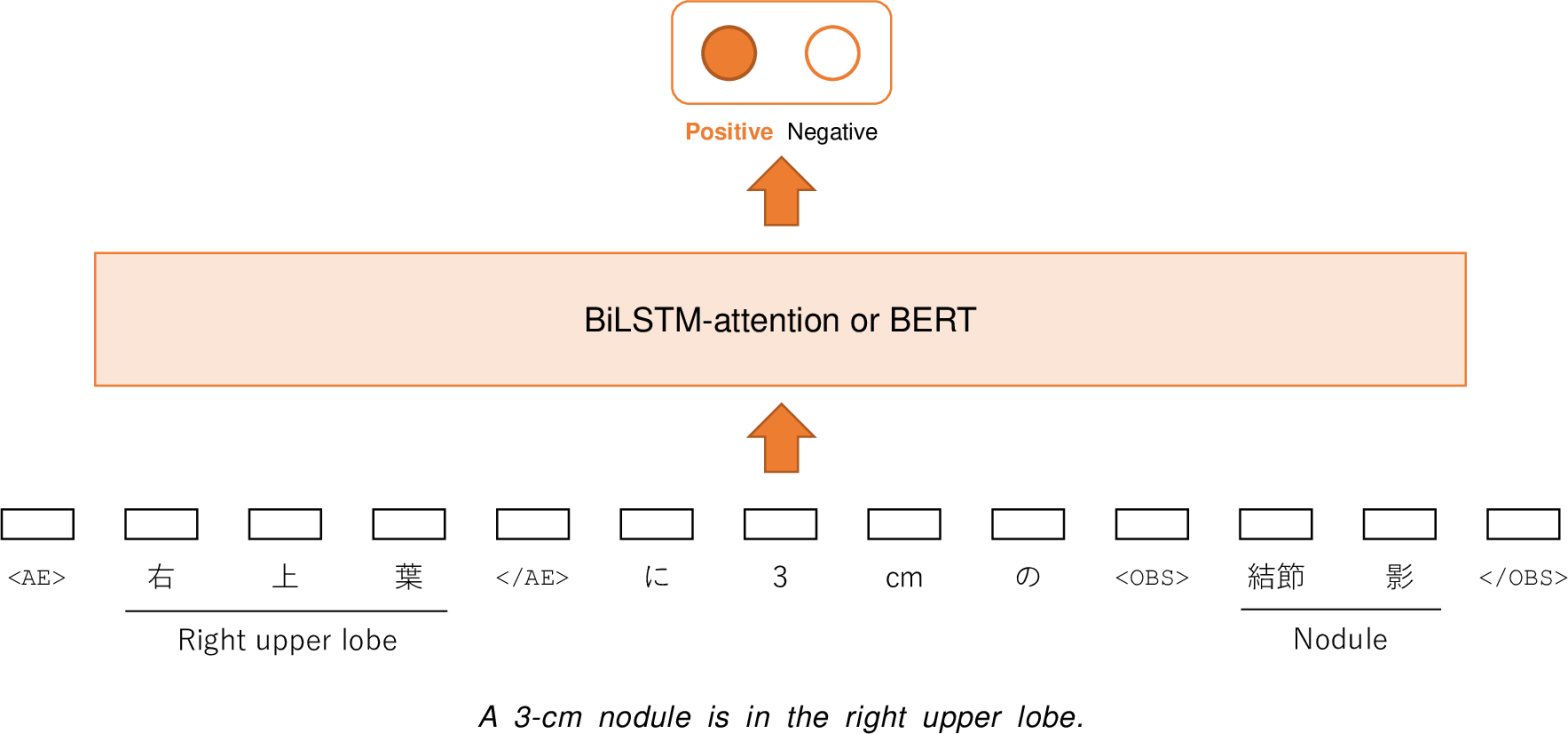
An illustration of the relation extraction module. BERT: Bidirectional Encoder Representations from Transformers; BiLSTM: bidirectional long short-term memory.

Next, we explain how we represented each relation candidate in a fixed-length sequence. Previous studies have introduced a method to add position indicator tokens to the input sequence to indicate the entity span of the pair in the sequence [36,37]. We expanded this method to allow the representation of the entity types. These position indicator tokens are referred to as “entity span tokens.” For example, the input sequence of the model representing the relation between an observation entity and an anatomical location modifier entity was represented as follows: “A 3 cm <OBS> nodule </OBS> is in the <AE> right upper lobe </AE>.” Here, “<OBS>,” “</OBS>,” “<AE>,” and “</AE>” are entity span tokens. Possible entity span tokens were appended to the vocabulary, and thus, an entity span token was treated as a single token. The input sequence containing 4 entity span tokens was fed into the model. A classification example is illustrated in Figure 5. All generated relation candidates were transformed into fixed-length sequences and fed into the model.

The BiLSTM attention model [38] and BERT model were compared. For the BiLSTM attention model, the output vector representation for classification was obtained from the weighted sum of the sequence vector representations. For the BERT model, the representation of the first “[CLS]” token for classification was used, which is a straightforward sequence classification tasks introduced by the original BERT.

### Experimental Settings

#### Data Set Splitting

A total of 540 annotated chest CT reports were divided into 3 groups: 378 reports for training, 54 reports for development, and 108 reports for testing. Similarly, a total of 500 annotated abdomen CT reports were divided into 3 groups: 350 reports for training, 50 reports for development, and 100 reports for testing. In total, 728 reports for training, 94 reports for development, and 208 reports for testing were prepared.

#### Parameter Optimization

For the BiLSTM-CRF model, a minibatch stochastic gradient descent with momentum was used, and the initial learning rate and momentum were set to 0.1 and 0.9, respectively. The learning rate was reduced when the *F*_1_-score of the development data set stopped improving. Learning rate decay and a gradient clipping of 5.0 were used. Dropout [39] was applied on both the input and output vectors of the BiLSTM model. A batch size of 16, a dropout rate of 0.1, a word embedding dimension of 100, and a hidden layer dimension of 512 were chosen. For the BERT model, BERT_BASE_ was used, which has 12 layers of transformer blocks, 768 hidden units, and 12 self-attention heads. The model was fine-tuned with the initial learning rate of 5 × 10^–5^, a batch size of 16, and training epochs of 10. The best hyperparameter setting was chosen using a development data set.

#### Domain Adaptation

Previous studies have reported that pretraining the domain corpora improved the model performance for various downstream tasks [27,40,41]. However, some studies have pointed out that domain adaptation (DA) leads to a degradation in model performance due to forgetting general domain knowledge [42,43]. To validate the effect of DA in our experiments, we evaluated the model performance with and without DA for both the entity extraction and relation extraction models.

For pretraining the word embeddings of the BiLSTM model with the general domain, Japanese Wikipedia articles [44] (12 million sentences) were used. For pretraining the word embeddings of the BiLSTM model with DA, 911,465 in-house radiology reports were used. We used word2vec (Mikolov et al [45]) for both tasks of pretraining the word embeddings.

For the BERT model, the publicly available pretrained Japanese BERT (Tohoku NLP Group and Tohoku University) [46] was first initialized. The model was pretrained using Japanese Wikipedia articles. The BERT_BASE_ subword tokenization model pretrained with whole word masking was chosen. For DA, continued pretraining using 911,465 in-house radiology reports for approximately 100,000 steps using a batch size of 256 was implemented.

#### Evaluation Metrics

To validate the capability of our system, we conducted 2 experiments. First, the performances of the deep learning modules were calculated. In this experiment, the mean scores were obtained over 5 runs with different parameter initializations to mitigate the effects of a random seed. For both the entity extraction and relation extraction, the *F*_1_-score was used for evaluation. For the entity extraction, entity-level *F*_1_-score was used as an evaluation metric, and the results were aggregated by microaveraging. Second, to validate that our information model encompassed clinical information in the reports, we measured the coverage with the following formula:


$$Coverage\left(\%\right)=\frac{B\text{-}tagged\;tokens\;+\;I\text{-}tagged\;tokens}{B\text{-}tagged\;tokens\;+\;I\text{-}tagged\;tokens\;+\;O\text{-}tagged\;tokens}$$

where B-tagged tokens and I-tagged tokens were annotated as entities represented in the IOB2 format [33], and O-tagged tokens as outside entities were not annotated. Following to the scope definition of our information model, the sentences that only contained information about the technique of the imaging test, the surgical procedures of the patients, and recommendations were excluded. Punctuations and stop words were also excluded from the calculation. The list of stop words is presented in Multimedia Appendix 2.

## Results

### Entity Extraction

Table 1 shows the performance metrics for the entity extraction model. The BiLSTM-CRF model with DA achieved a microaveraged *F*_1_-score of 96.1%. In our experiments, the BiLSTM-CRF model with DA achieved the best performance of all the microaveraged scores. For the BERT model, concatenating the CRF layer to the output of the BERT improved the mean *F*_1_-scores with and without DA. Given that the BiLSTM-CRF model with DA yielded the highest mean *F*_1_-score, it was used as the entity extraction module for our system and was used for the remaining experiments.

**Table 1.** Comparison of entity extraction models using mean *F*_1_-scores.

Model	Without DA^a^, mean *F*_1_-score (%)	With DA, mean *F*_1_-score (%)
BiLSTM^b^	95.2	*96.1^c^*
BERT^d^	94.8	95.2
BERT-CRF^e^	95.1	95.4

a DA: domain adaptation.

b BiLSTM: bidirectional long short-term memory.

c The best performance is italicized.

d BERT: Bidirectional Encoder Representations from Transformers.

e CRF: conditional random field.

The detailed performance of BiLSTM-CRF model with DA is shown in Table 2. In the test set using chest and abdomen reports, the *F*_1_-scores of observation, clinical finding, anatomical location modifier, certainty modifier, and size modifier entities were over 95%, whereas the change modifier and characteristics modifier entities had lower *F*_1_-scores than the other entities. Table 2 also shows that the test set of abdomen reports had a 0.5% higher *F*_1_-score than the chest reports. On the test set of abdomen reports, the clinical finding and change modifier entities achieved better *F*_1_-scores than the chest reports, with an increase of 2.9% and 2.5%, respectively. Conversely, the observation and characteristics modifier entities using the test set of chest reports obtained better *F*_1_-scores than the abdominal reports, with an increase of 1.0% and 2.6%, respectively.

**Table 2.** Comparison of the results of the entity extraction model for the test set of chest and abdomen reports.

Entity type	Chest reports, *F*_1_-score (%)	Abdomen reports, *F*_1_-score (%)	Chest and abdomen reports, *F*_1_-score (%)
Observation	96.1	95.1	95.6
Clinical finding	94.2	97.1	96.1
Anatomical location modifier	96.3	96.3	96.3
Certainty modifier	98.6	99.1	98.9
Change modifier	90.5	93.0	91.5
Characteristics modifier	89.5	86.9	88.5
Size modifier	98.7	98.7	98.7
Microaverage	95.8	96.3	96.1

### Relation Extraction

The performances of the relation extraction models were compared. In this experiment, to focus on evaluating the relation extraction module, human-annotated entities were used for the input of each model. Table 3 shows the comparisons of the performance of the relation extraction models. A microaveraged *F*_1_-score of 95.6% was achieved for the BiLSTM attention model with DA and 97.6% for the BERT model with DA, which indicated that both classification models could achieve a satisfactory performance for relation extraction. Pretraining with domain corpora improved the performance of both relation models. In contrast to the experimental results of the entity extraction models, the BERT model outperformed the BiLSTM attention model by 2.0% in the *F*_1_-score.

**Table 3.** *F*_1_-score of the relation extraction models.

Model	Without DA^a^, microaveraged *F*_1_-score (%)	With DA, microaveraging *F*_1_-score (%)
BiLSTM^b^	95.5	*95*.6^c^
BERT^d^	97.2	*97*.6

a DA: domain adaptation.

b BiLSTM: bidirectional long short-term memory.

c The best performance is italicized.

d BERT: Bidirectional Encoder Representations from Transformers.

The performance difference between the chest and abdomen reports was also compared (Table 4). The *F*_1_-scores of the modifier relation were almost the same for the chest reports and abdomen reports, whereas the evidence relation was 6.3% lower in the abdomen reports than the chest reports.

**Table 4.** Comparison of the results of the relation extraction model for the test set of chest and abdomen reports.

Relation type and entity type		Chest reports, *F*_1_-score (%)	Abdomen reports, *F*_1_-score (%)	Chest and abdomen reports, *F*_1_-score (%)
**Modifier relation**				
	Anatomical location	97.9	97.6	97.6
	Certainty	99.4	99.5	99.4
	Change	95.4	95.0	95.1
	Characteristics	95.1	96.5	95.7
	Size	99.1	98.0	98.8
**Evidence relation**				
	Clinical finding	96.7	90.4	94.9
Microaverage		97.7	97.4	97.6

### Our 2-Stage System

To evaluate the performance of the entire pipeline of our system, the performance of the relation extraction module using the output of the entity extraction module was examined. According to the experimental results, the BiLSTM-CRF and BERT models were used for the entity extraction model and relation extraction model, respectively. Table 5 shows that the performance of the 2-stage system obtained an overall *F*_1_-score of 91.9%. The overall *F*_1_-score was 5.7% lower than the results using the human-annotated entities, as shown in Table 3. This decrease is reasonable since the misclassification of entity extraction is fed into the relation extraction model in this experiment.

**Table 5.** The *F*_1_-score of our 2-stage system.

Relation type and entity type		2-Stage system, *F*_1_-score (%)
**Modifier relation**		
	Anatomical location	92.8
	Certainty	96.3
	Change	81.4
	Characteristics	84.7
	Size	94.6
**Evidence relation**		
	Clinical findings	87.1
Microaverage		91.9

### Coverage of Clinical Entities

The test set of reports contained an average of 11.9 sentences. An average of 1.0 (8.4%) out of 11.9 sentences about the technique of the imaging test, the surgical procedures of the patients, and recommendations were excluded from the calculation. Table 6 shows the coverage of clinical entities with our information model. The coverage of the clinical entities across entire sequence was 70.2% (7050/10,036). We observed that 96.2% (6595/6853) of tokens were annotated when punctuations and stop words were excluded from the sequences.

**Table 6.** Coverage of the clinical entities with our information model.

Token scope	Annotated tokens , n/N (%)
Entire sequence	7050/10,036 (70.2)
Without punctuations and stop words	6595/6853 (96.2)

### Error Analysis

A quantitative error analysis was further performed to understand our 2-stage system. For the entity extraction module, we found that the entity mentions that rarely occurred in our corpus were likely missed. To evaluate this empirically, 2 additional test sets were used.

1. Major test set: entity mentions that occurred multiple times in the training set
2. Minor test set: entity mentions that only occured once or did not occur in the training set

Table 7 shows the comparison of the result of the major and minor test sets with the original test set (Table 3). In the major test set, the *F*_1_-score of the overall entities was improved by 2.1% (from 96.1% to 98.2%). This increase was also observed in the individual entities except for the size modifier entity. However, the *F*_1_-score of the overall entities was markedly decreased by 9% in the minor test set. This was expected as the deep learning model struggled to predict the samples that were rare or unseen in the training set. Another reason for this difference may be the difficulty in determining the appropriate entities for the minor mentions. We observed that annotation disagreements during the adjudication process occurred more frequently for the minor mentions than the major mentions. Interestingly, we found that the size modifier was robust to the minor entity mentions. The simplicity of these entity mentions, such as “5 cm” and “30×14 mm,” may have contributed to the result. Our analysis shows that the entity extraction module could extract frequent entity mentions in the training set accurately; however, there remains much room for improvement regarding rare or unseen terms in the training set.

**Table 7.** Error analysis.

Entity type	Original test set, *F*_1_-score (%)	Major test set		Minor test set	
		*F*_1_-score (%)	Difference from the original test set	*F*_1_-score (%)	Difference from the original test set
Observation	95.6	97.9	+2.3	82.0	–13.6
Clinical finding	96.1	97.9	+1.9	87.8	–8.2
Anatomical location modifier	96.3	98.7	+2.4	89.6	–6.7
Certainty modifier	98.9	99.3	+0.4	80.5	–18.4
Change modifier	91.5	93.5	+2.0	89.0	–2.5
Characteristics modifier	88.5	95.5	+7.1	61.5	–26.9
Size modifier	98.7	98.	–0.3	98.2	–0.6
Microaverage	96.1	98.2	+2.1	87.1	–9.0

To decrease the ratio of rare or unseen terms in the test set, more samples would be required in the training set. However, it is inefficient to sample reports randomly to improve the overall performance. For an efficient sampling strategy, active learning [47,48] may be a promising approach that allows for the selective sampling of reports in the current module.

The performance of the entity extraction and relation extraction modules were compared using the test set of chest and abdomen reports, respectively. For the entity extraction, the *F*_1_-score of the clinical finding entities in the test set for the abdomen reports was 2.9% better than that of the chest reports. In the abdomen reports, it was often written using terms such as “肝臓 : n.p. (Liver: n.p.)” when there were no particular findings for a specific organ. This simple expression, “n.p.,” constituted 66.2% of the clinical finding entities in the test set of the abdomen reports, which substantially impacted the performance.

**Figure 6.**
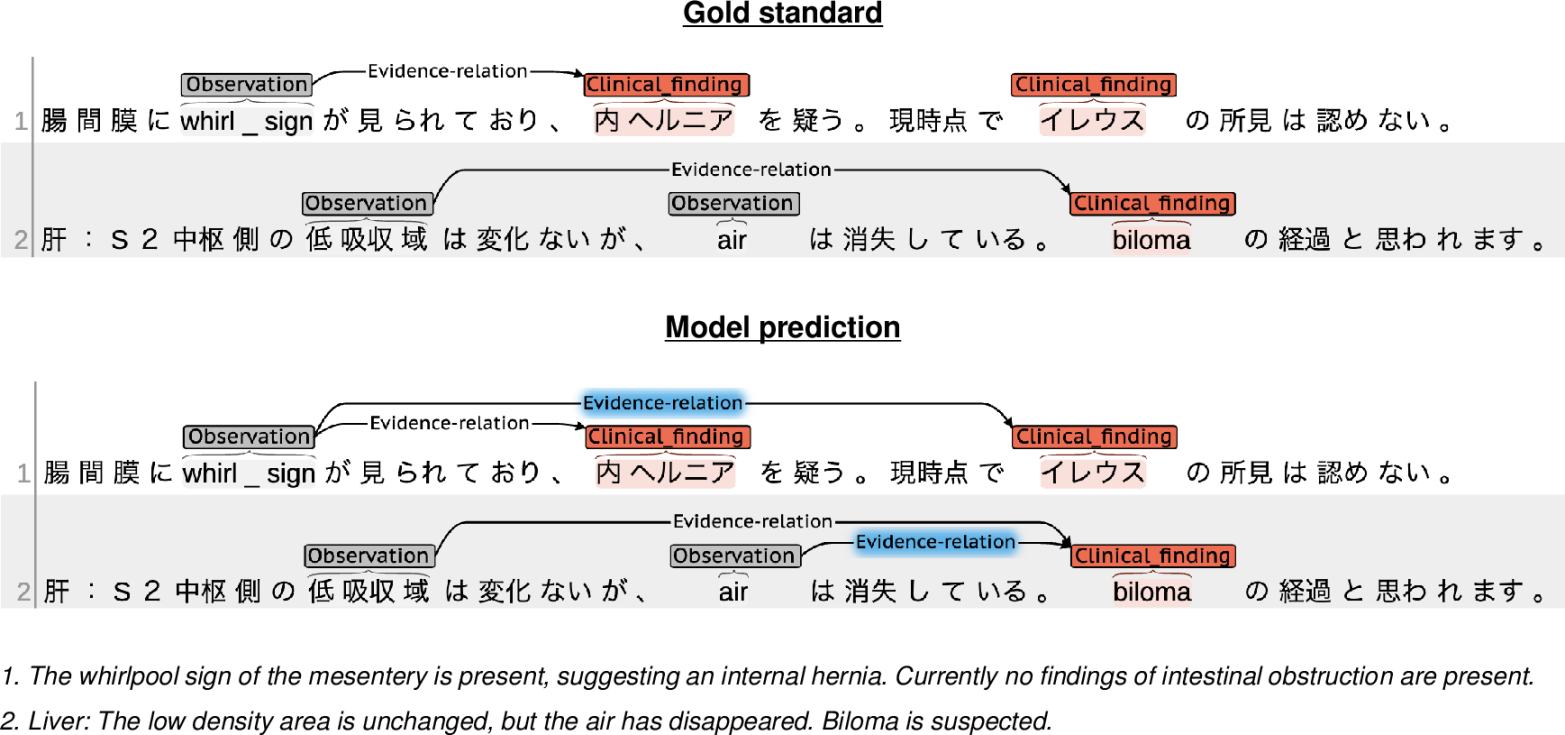
Misclassification examples of the relation extraction model (blue highlighted relations are examples of false positives).

The overall performance of the relation extraction module demonstrated excellent performance on the test set for both the chest and abdomen reports. However, the *F*_1_-score for the evidence relation between the observation and clinical finding entities was 6.3% lower on the test set of the abdomen reports than that of the chest reports. We found a few examples where the observations and clinical findings were clinically related; however, we could not determine if the observation was the diagnostic basis for the finding. The first example shown in Figure 6 indicates that the “whirlpool sign” was the observation for the diagnostic basis of an “intestinal obstruction (イレウス),” whereas no observation was found for the diagnostic basis of an “intestinal obstruction (イレウス).” Even though a “whirlpool sign” was clinically related to an “intestinal obstruction (イレウス),” the evidence relation cannot be derived from this example. However, our model misclassified this as a positive example of the evidence relation. In the second example, annotators did not assign the evidence relation between “air” and “biloma,” since they considered that the “air” has already disappeared. However, we discussed that the clinical finding of “biloma” was actually derived from the evidence of an unchanged “low density area (低吸収域)” and disappeared “air.” Thus, the model prediction was more preferable than the gold standard. To derive the diagnostic basis, it is preferable to consider information about the observation and its modifying entities.

## Discussion

### Principal Findings

Table 3 shows the performance of the entity extraction model, which yielded a microaveraged *F*_1_-score of 96.1%. The *F*_1_-scores of the observation entity and the clinical finding entity were 95.6% and 96.1%, respectively. These superior performances are desirable for our system since the observation and clinical finding entities are principal components of our information model. Moreover, Table 5 shows that the modifier relation with the certainty entity also had superior performance. These results suggest that our system will be applicable for practical secondary uses, such as a query-based case retrieval system [49]. However, to reuse radiology reports for various clinical applications, improvements in extracting the change modifier and characteristics modifier would also be required.

### BiLSTM Versus BERT

Table 3 shows that the BiLSTM-based model achieved better performance than the BERT-based model in the entity extraction task, whereas Table 5 shows that the BERT-based model outperformed the BiLSTM-based model in the relation extraction task. We considered that the differences between entity and relation extractions might be due to their task characteristics. Local neighborhood information and the representation of the token itself are considered important in the entity extraction task, whereas more global context information is required in the relation extraction task, especially for long-distance relations. Due to their attention mechanism, BERT and other transformer-based models are capable of learning long-range dependencies [50], which probably contributed to the superiority of the BERT model in the relation extraction task.

### DA Performance

Tables1  3 show the comparison results of the model performances with and without DA for each task. These results indicate that DA is beneficial for performance improvement, regardless of the architecture of the model. Since our system focuses on extracting information from radiology reports, we consider that the problem of forgetting general domain knowledge to be outside the scope of this study.

### Coverage of Clinical Entities

The coverage of the clinical entities with our information model was calculated. Sentences about the technique of the imaging test, the surgical procedures of the patients, and recommendations were excluded from the calculation, as such information was outside of the scope of our information model. Punctations and stop words were also excluded from the calculation. A total of 96.2% (6595/6853) of tokens were annotated, which indicates that our information model covered most of the clinical information in the reports.

### Limitations

This study has a limitation in terms of generalizability, since we only used 1 institutional data set for evaluation. More data sets outside our institution would be needed to ensure generalizability. Although we validated the capability of our system using only chest and abdomen CT reports, fine-tuning of the deep learning models with reports for other body parts and modalities would be required for various secondary uses.

Furthermore, we are aware that there is still a gap to bridge to reuse radiology reports for various applications. As reports usually contain misspellings, abbreviations, and nonstandard terminologies, we believe that term normalization techniques [51,52] would be needed for clinical applications.

### Conclusions

This study developed a 2-stage system to extract structured clinical information from radiology reports. First, we developed an information model and annotated in-house chest and abdomen CT reports. Second, we trained and evaluated the performance of 2 deep learning modules. The microaveraged *F*_1_-scores of our best model for entity extraction and relation extraction were 96.1% and 97.4%, respectively. The entire pipeline of our system achieved a microaveraged *F*_1_-score of 91.9%. Finally, we measured the ratio of annotated entities in the reports. The coverage of the clinical information in the reports was 96.2% (6595/6853). To reuse radiology reports, future studies should focus on term normalization. We also plan to develop a platform that allows us to evaluate the generalizability of our system using reports from outside of our institution.

## Supplementary material

10.2196/49041 Multimedia Appendix 1 The number of annotated entities and relations.

10.2196/49041 Multimedia Appendix 2 The list of stop words in Japanese.

## Abbreviations

BERT: Bidirectional Encoder Representations from Transformers
BiLSTM: bidirectional long short-term memory
CRF: conditional random field
CT: computed tomography
DA: domain adaptation
IAA: interannotator agreement
IE: information extraction
NER: named entity recognition
NLP: natural language processing

## References

[1] European Society of Radiology (ESR) (2018). ESR paper on structured reporting in radiology. Insights Imaging.

[2] Ganeshan D, Duong PAT, Probyn L, Lenchik L, McArthur TA, Retrouvey M (2018). Structured reporting in radiology. Acad Radiol.

[3] Demner-Fushman D, Chapman WW, McDonald CJ (2009). What can natural language processing do for clinical decision support?. J Biomed Inform.

[4] Jensen PB, Jensen LJ, Brunak S (2012). Mining electronic health records: towards better research applications and clinical care. Nat Rev Genet.

[5] Meystre SM, Savova GK, Kipper-Schuler KC, Hurdle JF (2008). Extracting information from textual documents in the electronic health record: a review of recent research. Yearb Med Inform.

[6] Sarawagi S (2008). Information extraction. Foundations and Trends in Databases.

[7] Small SG, Medsker L (2014). Review of information extraction technologies and applications. Neural Comput Appl.

[8] Zeng QT, Goryachev S, Weiss S, Sordo M, Murphy SN, Lazarus R (2006). Extracting principal diagnosis, co-morbidity and smoking status for asthma research: evaluation of a natural language processing system. BMC Med Inform Decis Mak.

[9] Savova GK, Masanz JJ, Ogren PV, Zheng J, Sohn S, Kipper-Schuler KC (2010). Mayo clinical Text Analysis and Knowledge Extraction System (cTAKES): architecture, component evaluation and applications. J Am Med Inform Assoc.

[10] Aronson AR (2001). Effective mapping of biomedical text to the UMLS Metathesaurus: the MetaMap program. Proc AMIA Symp.

[11] Friedman C, Hripcsak G, DuMouchel W, Johnson SB, Clayton PD (1995). Natural language processing in an operational clinical information system. Nat Lang Eng.

[12] Johnson DB, Taira RK, Cardenas AF, Aberle DR (1997). Extracting information from free text radiology reports. Int J Digit Libr.

[13] Lindberg DA, Humphreys BL, McCray AT (1993). The Unified Medical Language System. Methods Inf Med.

[14] Taira RK, Soderland SG (1999). A statistical natural language processor for medical reports. Proc AMIA Symp.

[15] Névéol A, Dalianis H, Velupillai S, Savova G, Zweigenbaum P (2018). Clinical natural language processing in languages other than English: opportunities and challenges. J Biomed Semantics.

[16] Spasic I, Nenadic G (2020). Clinical text data in machine learning: systematic review. JMIR Med Inform.

[17] Wang Y, Wang L, Rastegar-Mojarad M, Moon S, Shen F, Afzal N (2018). Clinical information extraction applications: a literature review. J Biomed Inform.

[18] Hassanpour S, Langlotz CP (2016). Information extraction from multi-institutional radiology reports. Artif Intell Med.

[19] Lafferty JD, McCallum A, Pereira FCN Conditional random fields: probabilistic models for segmenting and labeling sequence data.

[20] Cornegruta S, Bakewell R, Withey S, Montana G Modelling radiological language with bidirectional long short-term memory networks.

[21] Graves A, Schmidhuber J (2005). Framewise phoneme classification with bidirectional LSTM and other neural network architectures. Neural Netw.

[22] Miao S, Xu T, Wu Y, Xie H, Wang J, Jing S (2018). Extraction of BI-RADS findings from breast ultrasound reports in Chinese using deep learning approaches. Int J Med Inform.

[23] Suárez-Paniagua V, Rivera Zavala RM, Segura-Bedmar I, Martínez P (2019). A two-stage deep learning approach for extracting entities and relationships from medical texts. J Biomed Inform.

[24] Zhang X, Zhang Y, Zhang Q, Ren Y, Qiu T, Ma J (2019). Extracting comprehensive clinical information for breast cancer using deep learning methods. Int J Med Inform.

[25] Xie Z, Yang Y, Wang M, Li M, Huang H, Zheng D (2019). Introducing information extraction to radiology information systems to improve the efficiency on reading reports. Methods Inf Med.

[26] Jain S, Agrawal A, Saporta A, Truong SQH, Duong DN, Bui T (2021). RadGraph: extracting clinical entities and relations from radiology reports.

[27] Sugimoto K, Takeda T, Oh JH, Wada S, Konishi S, Yamahata A (2021). Extracting clinical terms from radiology reports with deep learning. J Biomed Inform.

[28] Cohen J (1960). A coefficient of agreement for nominal scales. Educ Psychol Meas.

[29] Landis JR, Koch GG (1977). The measurement of observer agreement for categorical data. Biometrics.

[30] Stenetorp P, Pyysalo S, Topić G, Ohta T, Ananiadou S, Tsujii J BRAT: a web-based tool for NLP-assisted text annotation. https://aclanthology.org/E12-2021.

[31] Li J, Sun A, Han J, Li C (2022). A survey on deep learning for named entity recognition. IEEE Trans Knowl Data Eng.

[32] Kudo T MeCab: yet another part-of-speech and morphological analyzer. GitHub.

[33] Sang EFTK, Veenstra J Representing text chunks. https://aclanthology.org/E99-1023.

[34] Lample G, Ballesteros M, Subramanian S, Kawakami K, Dyer C Neural architectures for named entity recognition.

[35] Devlin J, Chang MW, Lee K, Toutanova K BERT: pre-training of deep bidirectional transformers for language understanding.

[36] Zhang D, Wang D (2015). Relation classification via recurrent neural network. arXiv.

[37] Zhou P, Shi W, Tian J, Qi Z, Li B, Hao H Attention-based bidirectional long short-term memory networks for relation classification.

[38] Bahdanau D, Cho K, Bengio Y (2014). Neural machine translation by jointly learning to align and translate. arXiv.

[39] Srivastava N, Hinton G, Krizhevsky A, Sutskever I, Salakhutdinov R (2014). Dropout: a simple way to prevent neural networks from overfitting. J Mach Learn Res.

[40] Jauregi Unanue I, Zare Borzeshi E, Piccardi M (2017). Recurrent neural networks with specialized word embeddings for health-domain named-entity recognition. J Biomed Inform.

[41] Gururangan S, Marasović A, Swayamdipta S, Lo K, Beltagy I, Downey D Don’t stop pretraining: adapt language models to domains and tasks.

[42] Wiese G, Weissenborn D, Neves M Neural domain adaptation for biomedical question answering.

[43] Thompson B, Gwinnup J, Khayrallah H, Duh K, Koehn P Overcoming catastrophic forgetting during domain adaptation of neural machine translation.

[44] (2023). Index of /jawiki/latest/: jawiki-latest-pages-articles.xml.bz2. Wikipedia.

[45] Mikolov T, Chen K, Corrado G, Dean J (2013). Efficient estimation of word representations in vector space. arXiv.

[46] Tohoku NLP Group, Tohoku University Pretrained Japanese BERT models. GitHub.

[47] Settles B (2009). Active learning literature survey. University of Wisconsin-Madison.

[48] Ren P, Xiao Y, Chang X, Huang PY, Li Z, Gupta BB (2021). A survey of deep active learning. ACM Comput Surv.

[49] Pons E, Braun LMM, Hunink MGM, Kors JA (2016). Natural language processing in radiology: a systematic review. Radiology.

[50] Vaswani A, Shazeer N, Parmar N, Uszkoreit J, Jones L, Gomez AN Attention is all you need.

[51] Leaman R, Islamaj Dogan R, Lu Z (2013). DNorm: disease name normalization with pairwise learning to rank. Bioinformatics.

[52] Leaman R, Lu Z (2016). TaggerOne: joint named entity recognition and normalization with semi-Markov models. Bioinformatics.

