# Supplementary material for: Extracting Clinical Information From Japanese Radiology Reports Using a 2-Stage Deep Learning Approach: Algorithm Development and Validation
Source: JMIR Med Inform. 2023 Nov 14;11:e49041. doi: 10.2196/49041 (PMC10686535; doi:10.2196/49041)
Supplement: Multimedia Appendix 1 [file medinform-v11-e49041-s001.pdf]

S1 Table

Number of entities in each dataset

**Entity type**	**train**	**dev**	**test**
Observation	4,731	590	1,351
Clinical finding	2,804	369	790
Anatomical location modifier	5,507	656	1,522
Certainty modifier	4,545	549	1,284
Change modifier	1,081	156	293
Characteristics modifier	857	113	280
Size modifier	442	73	127
**All**	**19,967**	**2,507**	**5,647**

S2 Table

Number of relations in each dataset

**Relation type**	**train**	**dev**	**test**
**Modifier relation**			
Anatomical location	7,659	879	2,218
Certainty	5,079	611	1,441
Change	1,274	181	366
Characteristics	859	114	292
Size	420	70	122
**Evidence relation**			
Clinical finding	1,838	191	576
**All**	**17,129**	**2,046**	**5,015**
