# Supplementary material for: Extracting Clinical Information From Japanese Radiology Reports Using a 2-Stage Deep Learning Approach: Algorithm Development and Validation
Source: JMIR Med Inform. 2023 Nov 14;11:e49041. doi: 10.2196/49041 (PMC10686535; doi:10.2196/49041)
Supplement: Multimedia Appendix 2 [file medinform-v11-e49041-s002.pdf]

Excluded stop words (in Japanese)

で, は, に, が, の, へ, に, て, な, を, と, も, （, ）, い, よう, ます, おり, られ, ：,;,*, ・, -, また, や, し, いる, まで, など, から, さ, れ, する, た, より, です, いずれ, および, による, にかけて, ／
